# Integration of Metabolomics and Transcriptomics Reveal the Mechanism Underlying Accumulation of Flavonols in Albino Tea Leaves

**DOI:** 10.3390/molecules27185792

**Published:** 2022-09-07

**Authors:** Qunfeng Zhang, Chunlei Li, Zixin Jiao, Jianyun Ruan, Mei-Ya Liu

**Affiliations:** 1 Key Laboratory for Plant Biology and Resource Application of Tea, Tea Research Institute, Chinese Academy of Agricultural Sciences, The Ministry of Agriculture, Hangzhou 310058, China; 2 Shandong Provincial University Laboratory for Protected Horticulture, Weifang University of Science and Technology, Shouguang 262700, China

**Keywords:** albino mutation, *Camellia sinensis*, flavonoids, metabolism

## Abstract

Albino tea plants (*Camellia sinensis*) have been reported to possess highly inhibited metabolism of flavonoids compared to regular green tea leaves, which improves the quality of the tea made from these leaves. However, the mechanisms underlying the metabolism of catechins and flavonols in albino tea leaves have not been well elucidated. In this study, we analyzed a time series of leaf samples in the greening process from albino to green in a thermosensitive leaf-color tea mutant using metabolomics and transcriptomics. The total content of polyphenols dramatically decreased, while flavonols (such as rutin) were highly accumulated in albino leaves compared to in green leaves. After treatment with increasing environment temperature, total polyphenols and catechins were increased in albino mutant tea leaves; however, flavonols (especially ortho-dihydroxylated B-rings such as rutin) were decreased. Meanwhile, weighted gene co-expression network analysis of RNA-seq data suggested that the accumulation of flavonols was highly correlated with genes related to reactive oxygen species scavenging. Histochemical localization further demonstrated that this specific accumulation of flavonols might be related to their biological functions in stress tolerance. These findings suggest that the temperature-stimulated accumulation of total polyphenols and catechins in albino mutant tea leaves was highly induced by enhanced photosynthesis and accumulation of its products, while the initial accumulation and temperature inhibition of flavonols in albino mutant tea leaves were associated with metabolism related to oxidative stress. In conclusion, our results indicate that the biosynthesis of flavonoids could be driven by many different factors, including antioxidation and carbon skeleton storage, under favorable and unfavorable circumstances, respectively. This work provides new insights into the drivers of flavonoid biosynthesis in albino tea leaves, which will further help to increase tea quality by improving cultivation measures.

## 1. Introduction

Flavonoids are the most important quality-related compounds in young tea plant shoots, comprising 20–40% of dry matter [1]. The characteristics of brewed tea, including color, taste, and aroma, are directly or indirectly associated with these flavonoid compounds. The predominant flavonoids in tea plants are catechins (flavan-3-ol), flavonols, flavonol glycosides, anthocyanin, and leucoanthocyanidin. Numerous reports show a wide variation in the levels and composition of phenolic compounds in teas from different locations, elevations and seasons, which is often attributed to differences in variety, temperature, irradiance, rainfall, and nutrient or water supply [2,3]. For example, accumulation of flavonoids is strictly genetically controlled in a spatial and temporal manner and in response to several biotic and abiotic factors [4]. However, subgroups of flavonoids in tea plants can be differentially affected [5,6]. The biosynthesis of flavonoids in tea plants has been intensively investigated at the biochemical, physiological, and genetic levels. Significant progress has been made in identifying structural genes involved in the tea phenylpropanoid/flavonoid pathway in recent years [7,8,9]. However, little is known about the factors driving flavonoid biosynthesis.

Flavonoids are the main regulators of plant growth and defense, and they are induced and biosynthesized as a result of a long-term natural selection and acclimatization process [10,11,12]. The main physiological functions of flavonoids in tea plants are scavenging reactive oxygen species and increasing tolerance for adaptation to environmental change, e.g., as antioxidants in photoprotection. The antioxidant activity of flavonoids is attributed to their reaction with free radicals as a hydrogen donor. However, flavonoids with different molecular structures show great differences in their antioxidant activity, which is highly correlated with the substituent positions and the number of hydroxyl groups on the B-rings. The more hydroxylation of the flavonol, the more hydrogen atoms can be provided for binding free radicals, and therefore the greater the antioxidant activity, e.g., the antioxidant activity of myricetin is higher than that of kaempferol. Moreover, tautomeric interconversions of ortho-dihydroxylated B-ring flavonoids make them more efficient at dissipating excess energy [13] and scavenging reactive oxygen species [14,15,16].

Albino tea leaves show enhanced levels of free amino acids and low polyphenol content, which improve the quality of prepared tea and impart a higher economic value, compared with non-albino varieties [17,18]. ‘Baiye 1′, a temperature-sensitive, chlorophyll-deficient mutant [17], is one of the most popular cultivars for green tea. The leaf color of ‘Baiye 1′ was yellowish-white when the ambient temperature was below 22 °C; however, it became green and barely distinguishable from the leaves of the normal tea plants when the ambient temperature was higher than 22 °C. Microarray methods [17], proteomic analysis [19], enzyme analysis [20] and structural analysis of chlorophyll [21] have all been used to study the correlation between the phenotype and principal quality components of ‘Baiye 1′ [18,20]. However, a systematic approach has seldom been used to study the accumulation and biosynthesis of flavonoids in albino tea plants.

Here, we tested the hypothesis that the metabolism and accumulation of flavonoids, particularly different branches of the pathway, should correlate highly with substrate accumulation and the potential function (antioxidation and/or carbon skeleton storage) of flavonoids in tea leaves. We therefore compared samples of tea leaves from a short-time-course temperature treatment using metabolomic analyses.

## 2. Materials and Methods

### 2.1. Plant Material and Treatment

The natural mutant of *Camellia sinensis* (L.) (‘*Baiye 1′*) was planted in pots at the Tea Research Institute, Chinese Academy of Agricultural Sciences (TRI, CAAS). Five-year-old cutting seedlings of biennial clone ‘Baiye 1′ were cultivated in improved soil (commercial growth medium consisting of perlite, vermiculite, and peat) in an environmentally controlled growth chamber. In March 2015, 40 pots of tea plants were treated at 15 °C (air temperature) for 30 days until all young shoots produced were albino; uniform young shoots (one terminal bud and two young leaves) were marked for further treatment. Subsequently, 30 pots of plants were treated at 25 °C and the other 10 pots remained at 15 °C. The two groups of plants were treated under the same conditions except for air temperature: relative humidity was maintained at 75–80%; photo/dark period was 14/10 h; light intensity was 200 μmol·m^−2^·s^−1^. Young shoots were sampled at 0 (T0), 3 (T1) and 6 (T2) days after transfer to 25 °C. The same shoots from plants treated at 15 °C were harvested as a control group at 6 days (T0-2). Randomly selected samples of marked young shoots comprising one bud with two leaves were collected, frozen in liquid nitrogen immediately and stored in a −70 °C ultra-refrigerator. The sampling was repeated six times from each treatment. Harvested samples were freeze-dried and then ground into a fine powder using a ball mill (M301, Retsch GmbH, Haan, Germany) prior to metabolites analysis.

### 2.2. Electron Microscopy Analysis

A transmission electron microscope (TEM) was used to observe the ultrastructure of chlorotic leaves. Leaf samples (approximately 1 mm^2^) were fixed in 2.5% glutaraldehyde solution overnight at 4 °C. Ultrathin sections of fixed leaves were cut, stained and viewed under a JEM-1230 transmission electron microscope at an accelerating voltage of 80 kV.

### 2.3. Quantitative Determination of Catechin, Chlorophylls, Carotenoids, Total Flavonols and Total Polyphenols Content

Catechins, chlorophylls and carotenoids were analyzed on a reverse-phase high-performance liquid chromatographic system (Waters 2695, Milford, MA, USA) coupled to a DAD detector (Waters 2998, Milford, MA, USA) as described by Zhang et al. [6]. Separations were performed using a C18 reverse-phase column (250 mm × 4.6 mm i.d., Luna 5 µm, Phenomenex, Torrance, CA, USA) as described by Zhang et al. [22]. Total flavonols levels were analyzed according the method described by Muir et al. [23]. Tea leaf powder (six biological replicates, each 22.5 mg) was hydrolyzed in 75% MeOH containing 1.2 M of HCl (+0.1% TBHQ as antioxidant) at 90–95 °C for 1 h. Released flavonoid aglycons were analyzed by HPLC (Waters Alliance e2695, Milford, MA, USA) coupled to a photodiode array detector (Waters 996 PDA, Milford, MA, USA). The concentration of total polyphenols was determined by spectrophotometry with ferrous tartrate [24]. One mL of the tea extract was transferred into a 25 mL volumetric flask to react with 5 mL dyeing solution (containing 3.6 × 10^−3^ MFeSO4 and 3.5 × 10^−3^ M potassium sodium tartrate, KNaC_4_H_4_O_6_), 4 mL distilled water and 15 mL buffer (0.067 M Na_2_HPO_4_ and 0.067 M KH_2_PO_4_). Absorbance at 540 nm of the reaction solution was determined in a 1 cm light-path cell. Quantification of catechin, chlorophylls, carotenoids, and total flavonols was performed using calibration curves with external standards.

### 2.4. Metabolome Analysis Using UPLC-Q-TOF/MS

The metabolites in a sample of young shoots were extracted using 75% (*v/v*) methanol and 1% formic acid as described by Zhang et al. [6]. After randomization, a 2-μL sample was injected for UPLC-Q-TOF/MS (Waters, UPLC/Xevo G2-S Q-TOF) and separated using a HSS T3 column. The mobile solutions were water with 0.1% formic acid (A) and acetonitrile containing 0.1% formic acid (B) with a gradient as previously described by Zhang et al. [6]. The column was kept at 40 °C and the flow rate was 0.4 mL/min. Mass spectra were acquired using electrospray (ESI) source over the range of *m/z* 100–1700 with both positive and negative ion modes. Drying gas temperature was 350 °C, cone gas flow 50 L/h; desolvation gas flow 800 L/h. Stability of proposed method was tested by performing repeated injections of solutions prepared from authentic reagents catechins and gallic acid (Sigma-Aldrich Co., St. Louis, MO, USA) in every 2 h. Quality control (QC) sample, which mixed by all samples with equal volume, has been analyzed alongside. Data preprocessing was performed using TransOmics software (Version 1.0; Waters). Metabolite peaks were assigned from accurate mass measurements using online metabolite databases (METLIN, http://metlin.scripps.edu/, accessed on 21 March 2022; MassBank, http://www.massbank.jp, accessed on 21 March 2022), and retention times were compared with data from published literature [6]. A matrix was exported for further statistical analyses. The unit variance, gives equal weight to each data value and allowing systematic changes with small variance to be more readily detected, was scaled for further statistical analysis using SIMCA-P (version 13.0, Umetrics, Umea, Sweden). Unsupervised principal component analysis (PCA) was run to obtain a general overview of the variance of metabolites, and supervised projection to latent structure discriminant analysis (PLS-DA) was performed to obtain information on differences in the metabolite composition of samples. The data of QC samples were removed after checked in a PCA analysis, in which there were little variations between all QC samples, indicating that the present metabolomics analysis is reliable. Variable importance in the projection (VIP) values of all data from the sevenfold cross-validated OPLS model were taken as a coefficient for metabolite selection. Those variables with VIP > 1.0 were considered relevant for group discrimination. After the multivariate approaches, the significance of each metabolite in group discrimination was further measured using the Student’s *t*-test (*p* < 0.01). Univariate statistics were performed by one-way ANOVA using SPSS (version 15.0, SPSS Inc., Chicago, IL, USA).

### 2.5. Metabolome Analysis Using GC-TOF/MS

Polar primary metabolites were extracted using an adapted form of the method reported by Mokochinski et al. [25]. Specifically, 200 mg of tea leaf powder (six biological replicates) was extracted using 1.4 mL 80% (*v/v*) cold methanol containing ribitol as an internal standard. After vortexing (10 s) and centrifugation (12,000 rpm, 10 min), 500 µL of the supernatant was mixed with 375 µL chloroform and 750 µL Milli-Q^®^ ultrapure water. Thereafter, 50 µL of the upper (polar) phase was dried using vacuum centrifugation. Automated derivatization of the metabolites, with methoxyamine and N-methyl-N-(trimethylsilyl)trifluoroacetamide, was performed using a CombiPAL pipetting–autosampler robot (CTC Analytics AG, Zwingen, Switzerland) mounted atop the gas chromatography unit. After completion of the derivatization, a series of alkanes were automatically added to each sample well. The extracts were analyzed using the gas chromatography (GC) mass spectrometry (MS) system, comprising an Agilent 6890 gas chromatograph (Agilent Technologies, Santa Clara, CA, USA) coupled to a Pegasus III TOF-MS instrument (Leco Instruments, Saint Joseph, MI, USA), with hard ionization at 70 eV. Chromatographic separation was conducted using a capillary column (30 m × 0.25 mm i.d., 0.25 μm; DB-5 column; Agilent Technologies, Santa Clara, CA, USA). The temperature was set at 85 °C for 2 min, with a subsequent temperature gradient of 15 °C per min until a final temperature of 360 °C was reached. Annotations were based on comparing both the spectra and the retention index (RI) to standard compounds previously analyzed using the same system, by referring to the mass spectral databases of the National Institute of Standards and Technology (NIST; Gaithersburg, MD, USA) and the Max Planck Institute (Gölm, Germany) [26]. After normalized with internal standard, the metabolite intensity data were processed as same method described in metabolome analysis using UPLC-Q-TOF/MS in this study.

### 2.6. RNA Isolation, cDNA Library Construction, Illumina Deep Sequencing and Data Processing

Total RNA was extracted using Trizol reagent (Invitrogen, Waltham, MA, USA) following the manufacturer’s protocol. RNA integrity was confirmed using a 2100 Bioanalyzer (Agilent Technologies). RNA samples for transcriptome analysis were prepared using an Illumina kit following the manufacturer’s recommendations. Fragments were purified using agarose gel electrophoresis and enriched by PCR amplification to create a cDNA library. Sequencing and data processing were performed according to the method described by Wang et al. [27].

### 2.7. Gene Co-Expression Network, Module Identification and Gene Edge Number

Construction of a gene co-expression network, followed by module detection of highly correlated genes, was inferred from differentially expressed genes (DEGs) using weighted gene co-expression network analysis (WGCNA), an R software package [28]. WGCNA network construction and module detection were conducted using default settings. A dynamic cut-tree algorithm was used for automatically and precisely identifying modules in a hierarchical clustering dendrogram [29]. The total number of edges for genes within each module was estimated using a WGCNA edge weight ≥0.5 as a cut-off. Networks were visualized using Cytoscape 3.4.0 [30].

### 2.8. Quantitative Reverse-Transcription PCR Analysis

Total RNA was isolated using an RNAplant_plus kit (Tiangen, China). cDNA was synthesized using a PrimeScript^TM^ RT reagent Kit (TaKaRa). Quantitative reverse-transcription PCR (qRT-PCR) was performed on an Applied Biosystems 7300 machine (Carlsbad, CA, USA). Primer pairs used for qRT-PCR are shown in Table S1, and *GAPDH* was used as a reference gene. For each target gene, triplicate reactions were performed. Relative transcript levels were calculated against that of the internal control *GAPDH* using the formula 2^−ΔΔCt^. All data are shown as the mean ± SD (*n* = 3).

### 2.9. Tissue Localization of Phenolic Compounds

Samples were prepared by standard freehand sectioning and stained with 1% (*w/v*) vanillin-HCl reagent to study the localization of phenolic compounds. Stained samples were observed under a microscope (XQT-2, COIC), and images of the sections were recorded before and after staining [31].

The localization of flavonoids was obtained by staining sections with NaturstoVreagenz A using confocal laser scanning microscopy (CLSM, Zeiss LSM 710 NLO) as described by Hutzler et al. [32].

## 3. Results

### 3.1. Phenotype and Ultra-Structure

**Figure 1:**
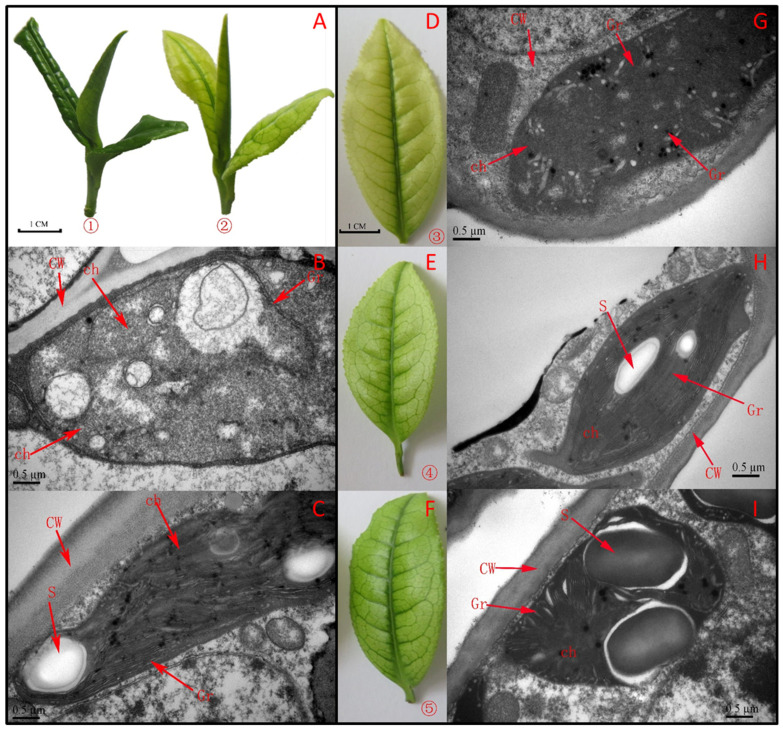
Phenotype (**A**,**D**–**F**) and ultrastructure (**B**,**C**,**G**–**I**) of ‘Baiye 1′ shoots cultivated for 30 days at 25 °C (**A①**,**C**) or 15 °C (**A②**,**B**), and for 0 (**D**,**G**), 3 (**E**,**H**) and 6 (**F**,**I**) days after starting greening. Note: CW, cell wall; Gr, grana; Ch, chloroplast; S, starch grains.

Leaves of ‘Baiye 1′ tea plants grown under 15 °C were albino, while those grown under 25 °C were green on both sides (Figure 1). Leaf color gradually turned green after treatment at 25 °C for 3 days, and much more color developed after treatment for 6 days. Chlorophyll *a* and chlorophyll *b* contents of albino leaves were only one third those of normal green leaves. By contrary, α-carotene, β-carotene and zeaxanthin contents were dramatically increased in albino leaves. After 3 days, contents of chlorophyll *a*, chlorophyll *b*, lutein and carotene in albino leaves increased significantly (*p* < 0.05) (Table 1). After 6 days, the contents of these pigment components were further increased.

Transmission electron microscopy analysis revealed apparent differences in ultrastructure between albino and green leaves (Figure 1). The chloroplasts of albino leaves were stagnant at the proplastid stage and did not develop a clear sheet membrane; they did not have grana structures but ubiquitous osmiophilic granules (Figure 1). The membrane system and cellular compartmentalization of chlorotic leaves were severely disrupted, and some chloroplasts showed cavitation. The thylakoid membrane structure, plastid and chloroplast granule were obviously restructured, consistent with the changes in leaf color.

### 3.2. Metabolic Changes of Catechins, Total Flavonol and Total Polyphenol Contents

Quantitative determination of catechins based on HPLC-DAD showed that the content of the catechin conjugates GC, EGC, EGCG, GCG and CG increased significantly under greening (Table 1). The content of flavonoids showed a significantly lower level in the albino leaves, which increased dramatically when the leaves regreening. In short, the content of flavonoids dramatically decreased, while flavonols (including myricetin, quercetin, and kaempferol glycosides) were highly accumulated in albino leaves than in green leaves. After treated with increasing environment temperature, total polyphenols and catechins are increased while flavonols are decreased in albino mutant tea leaves.

### 3.3. Overview of Metabolomic Profiling

**Figure 2:**
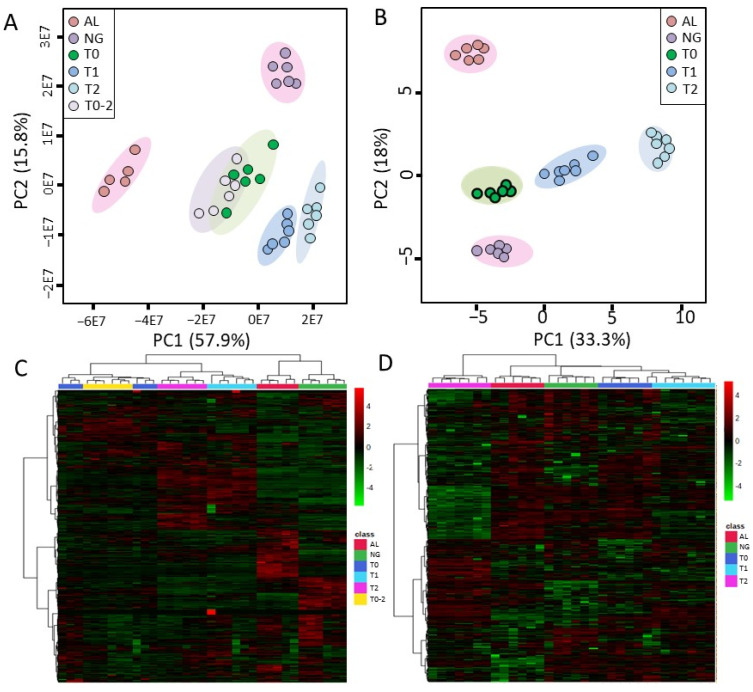
PCA score plot (**A**,**B**) and heatmap (**C**,**D**) of tea samples based on the relative variation of all compounds detected by the LCMS-semipolar platform (**A**,**C**) and all compounds detected by the GCMS-polar platform (**B**,**D**) using tea leaves cultivated for 30 days at 25 °C (NG) or 15 °C (AL), and for 0 (T0), 3 (T1) and 6 (T2) days after starting greening. The same shoots from plants treated at 15 °C were harvested as the control group at day 6 (T0-2).

We detected 1215 and 9403 (3889 positive and 5514 negative) compounds and independent mass spectral peaks by GC MS and UPLC-Q-TOF MS, respectively. Multivariate statistical analysis of the matrix obtained from the raw data revealed significant differences in metabolic profiles between albino (AL) and normal green (NG) samples and significant metabolic differences in tea leaves at different stages (greening from albino leaves). The PCA score plots (Figure 2A,B) and heatmaps (Figure 2C,D) from both the GC and LC platforms indicated the differences and clustering between these samples well. With the first two principal components of the PCA model, 73.7% (UPLC-Q-TOF MS) and 51.3% (GC/GC) of all variation could be explained, with predictive accuracy of 78% and 74%, respectively. With PCA and cluster analysis, the albino leaves and the normal green leaves showed significant differences. However, the samples from T0 and T0-2 were very similar in both the PCA score plot and the clustering and heatmap, compared with the large difference between T0 and T1 samples, indicating that normal growth of untreated tea shoots does not affect the metabolism in leaves.

### 3.4. Overview of Transcriptomic Analysis

In total, 9137 differentially expressed genes (DEGs) were selected between the albino (AL) and normal green (NG) tea leaves, T0 and T1 time points, and T1 and T2 time points for bioinformatics analysis. Functional classification of these DEGs using Gene Ontology (GO) showed that the expression levels of genes related to basic physiological metabolism were downregulated, while the transcription of stress response related genes were upregulated in the albino leaves (Table S2, AL and T0). The GO terms included: chloroplast thylakoid membrane, storage vacuole, and response to oxidative stress. Based on enrichment analysis using KEGG (Kyoto Encyclopedia of Genes and Genomes), we found the main differences between T0 and T1 and T2 resulted from changes in energy-related genes and secondary metabolic pathways (Table S3). These include genes/pathways related to photosynthesis, starch and sucrose metabolism, flavonoid biosynthesis, and anthocyanin biosynthesis.

### 3.5. Accumulation of Carbohydrates in Albino Shoots

Different metabolites, as measured by the GCMS-polar platform, identified between the different groups were selected on the basis of VIP > 1, P (corr) > 0.8 and Fold > 2 in the PLS-DA statistical model, and the various compounds are shown in Table 2, Table 3 and Table S4. We analyzed the metabolites using a meta-analysis for enrichment (Figure S1). The main differences in metabolic processes were in flavonol biosynthesis, tryptophan metabolism, citric acid cycle, caffeine metabolism, arginine and proline metabolism, amino sugar metabolism, and starch and sucrose metabolism (Figure S2). These metabolic processes are mainly involved downstream of photosynthetic and energy metabolism, as well as C assimilation, the supply of carbon skeleton, the use of N nutrition and other processes (Figure S2). The contents of carbohydrates, such as arabinopyranose, fructose and glucose, were significantly increased under greening process, while maltose content was significantly decreased (Table 3). Similar to carbohydrates, organic acids showed variable responses. The contents of most amino acids, such as glutamine and theanine, were significantly lower at both T1 and T2 compared with T0.

### 3.6. Metabolic Changes of Flavanols in Albino Shoots

As shown in Table 2, the contents of the various catechins analyzed were significantly and rapidly decreased in albino tea leaves (AL) when compared to normal green leaves (NG), while greening resulted in marked changes in secondary metabolite composition. Most flavonoids, such as epigallocatechin 3-cinnamate, cyanidin 3-rutinoside, and gladiatoside, were highly induced in albino tea leaves by greening. However, most flavonols analyzed showed high levels at T0. For example, the level of quercetin 3-rutinoside was 3.63-fold higher at T0 than in leaves of the T2 group. Notably, the compounds with significant difference indicated by multivariate analysis (LC-MS) the same as those with difference by quantification (LC-DAD).

### 3.7. Expression of Gene Related to Flavonoids Metabolic in Albino Shoots

**Figure 3:**
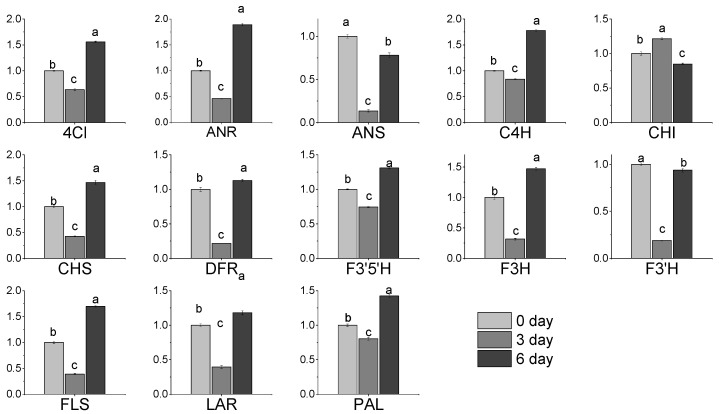
Expression of genes (means ± SD) related to flavonoid metabolism in albino (0 day) and green tea leaves of ‘Baiye 1′. 4Cl, 4-coumarate-CoA ligase; ANR, anthocyanidin reductase; ANS, anthocyanidin synthase; C4H, cinnamate 4-hydroxylase; F3′H, flavonoid-3′-hydroxylase; PAL, phenylalanine ammonia-lyase; CHI, chalcone isomerase; CHS, chalcone synthase; F3H, flavonoid-3-hydroxylase; LAR, leucoanthocyanidin reductase; FLS, flavonol synthase; DFR, dihydroflavonol-4-reductase; F3′5′H, flavonoid-3′, 5′-hydroxylase (*n* = 3). Different letters (a, b, c) above the bars indicate significant (*p* < 0.01) differences among the three shading treatments.

**Figure 4:**
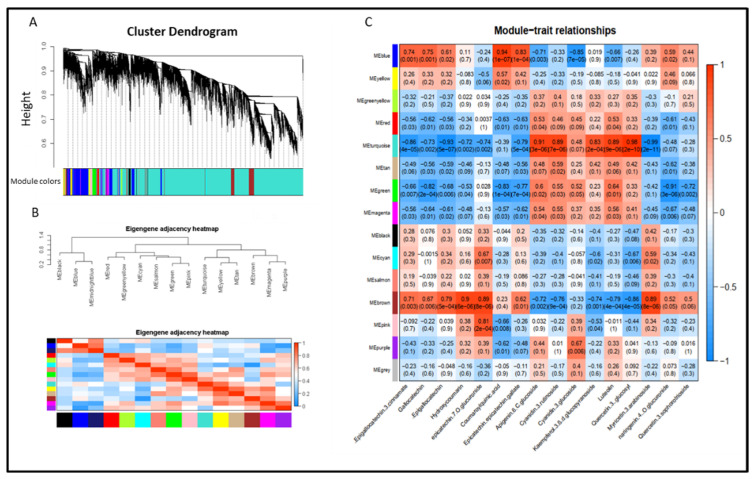
Gene modules (**A**) identified by weighted gene co-expression network analysis (WGCNA). Relationships of consensus module genes (**B**) and content of catechins and flavonols (**C**). The module name is shown on the left side of each cell. Numbers in the table report the correlations of the corresponding module eigengenes and contents, with the *p* values printed below the correlations in parentheses. The table is color coded by correlation according to the color legend. Intensity and direction of correlations are indicated on the right side of the heatmap (red, positively correlated; blue, negatively correlated).

We quantified the expression of key genes in the flavonoid pathway using qRT-PCR (Figure 3). Expression of all genes, except *CHI, ANS* and *F3′H* showed the lowest levels at 3 (T1) days and the highest levels at 6 (T2) days of cultivation under 25 °C after transfer from 15 °C conditions. To investigate genes related to flavonoid levels, we constructed co-expression modules using WGCNA. Fifteen WGCNA modules were identified among the DEGs. Module–trait correlation analysis indicated that the “blue” and “brown” modules were positively correlated with catechin content in tea plants (r = 0.74, *p* = 0.001; r = 0.71, *p* = 0.003; respectively) (Figure 4). Meanwhile, the “turquoise” modules were significantly positively correlated with flavonol content (r = 0.91, *p* = 3.0 × 10^−6^). By annotation of genes using GO terms, we found that genes in the “brown” and “turquoise” modules were associated with GO terms related to photosynthesis, metabolism, and defense response. To identify candidate genes controlling initiation and progression of flavonol metabolism, we analyzed the edge number for each node of the co-expression modules. For three WGCNA modules positively correlated with flavonols and catechins, eight hub genes were identified (Table 4). Among them, two photosynthesis-related genes (CSS0026029 and CSS0025971) and three carbohydrate transport genes, starch synthase (CSS0032826 and CSS0004941) and ADP glucose pyrophosphorylase (CSS0035261), were highly connected with other genes in the “brown” and “blue” modules. In the “turquoise” module, two genes related to defense mechanisms (CSS0040654) and response to oxidative stress (CSS0009596 and CSS0019730) were highly connected with other genes.

### 3.8. Intracellular Localization of Flavonoids

**Figure 5:**
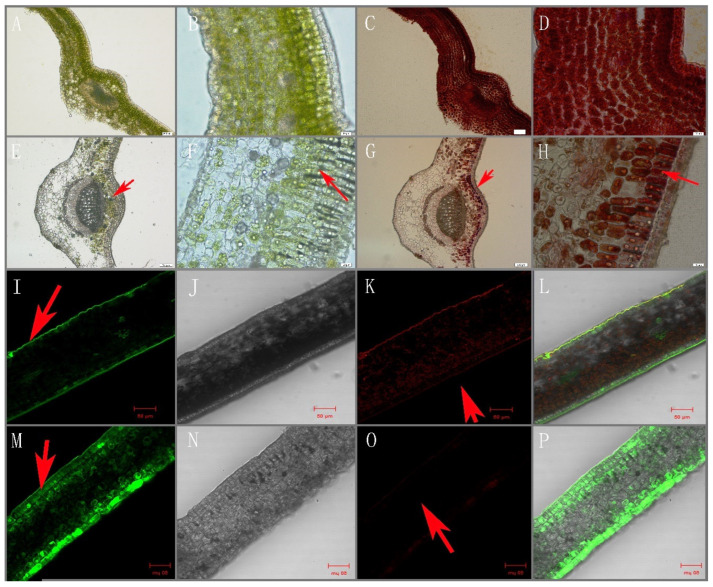
Distribution of catechin and dihydroxy flavonol in albino and green tea leaves of ‘Baiye 1′ cultivated for 30 days at 25 °C (NG) or 15 °C (AL). Note: (**A**–**D**) normal green leaf; (**E**–**H**) albino leaf; (**A**,**E**,**C**,**G**) no staining; (**B**,**D**,**F**,**H**) stained with 1% (*w/v*) vanillin-HCl reagent, red arrows marked the location of chlorophyll (**E**,**F**) and catechins (**G**,**H**). Tissue localization of flavonoids by confocal laser scanning microscopy (staining with NaturstoVreagenz **A**). Red signal, chlorophyll; green fluorescent signal. (**I**–**L**) Normal green leaves, and (**M**–**P**) albino leaves of baiye1, red arrows marked the location of flavonol (**I**,**M**) and chlorophyll (**K**,**O**).

The location of polyphenols was consistent with that of chloroplasts, which were mainly located in the palisade tissue around the vein and vascular bundle. However, the location of flavonols was significantly different from those flavonoids, especially in albino leaves. Moreover, flavonols in normal green leaves were mainly concentrated in the epidermis (Figure 5 green fluorescence signal), while flavonols in albino leaves were diffused in tissues and widely distributed in mesophyll cells (Figure 5 fluorescence signal).

## 4. Discussion

### 4.1. Temperature-Stimulated Accumulation of Total Polyphenols and Catechins in Albino Mutant Tea Leaves Is Highly Induced by Enhanced Photosynthesis and Accumulation of Its Products

Previous studies have revealed that dramatic changes in metabolism occur in albino tea leaves compared with regular green tea leaves [33]. In particular, the flavonoid content is decreased while the amino acid content is increased notably, indicating that both carbon and nitrogen metabolism have undergone significant changes [18,20]. In the present study, the total contents of catechins and polyphenols in albino leaves were lower than those in normal green leaves. We also found that photosynthesis, photosystems I and II and oxidative phosphorylation were suppressed at the transcriptional level and the expression levels of genes related to flavonoid metabolism were downregulated significantly in albino leaves. Such findings suggest that flavonoid biosynthesis was strongly stimulated under greening process. Meanwhile, the main secondary metabolic pathways, such as PAL (key step of flavonoid biosynthesis), which are supplied by C skeleton metabolism, were also seriously inhibited in albino leaves. Previous studies have suggested that the energy metabolism of albino leaves plays an important role in albinism [20], and that the pathways of catechin biosynthesis is severely inhibited in albino leaves [18,19].

**Figure 6:**
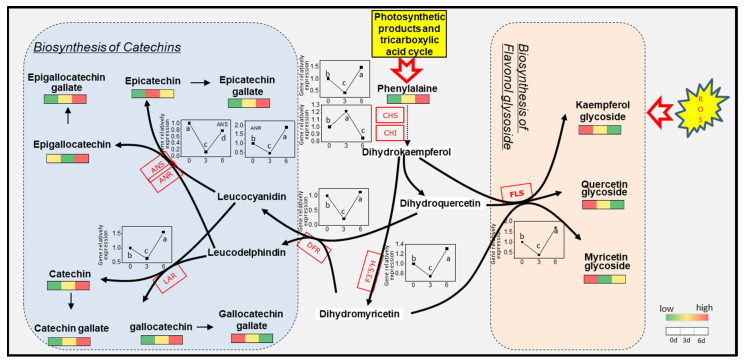
Schematic presentation of phenylpropanoid/flavonoid pathway in albino tea leaves of ‘Baiye 1′. ANS, anthocyanidin synthase; ANR, anthocyanidin reductase; LAR, leucoanthocyanidin reduc-tase; DFR, dihydroflavonol-4-reductase; CHS, chalcone synthase; CHI, chalcone isomerase; FLS, flavonol synthase; F3′5′H, flavonoid-3′, 5′-hydroxylase (*n* = 3). Different letters (a, b, c, d) above the bars indicate significant (*p* < 0.01) differences among the three shading treatments.

However, under the greening process, the contents of most flavonoids increased in the T1 and T2 shoots as compared with the T0 shoots (Table 1 and Table 2). Increasing flavonoid contents were accompanied by higher expression levels of most genes (except *CHI*) involved in the pathway starting from phenylalanine to these metabolites at T2, as well as the genes involved in the upstream shikimic acid pathway leading to the biosynthesis of Phe (Figure 6). Considering that cells capture their own cytoplasm and organelles and consume them in lysosomes under carbon starvation [34], for example, the autophagic degradation of chloroplasts is particularly activated in leaves under sugar-depleted conditions [35], one explanation for such stimulation lies in the restructuring of the chloroplast, which is the site of flavonoid biosynthesis [36]. Furthermore, the induced accumulation of flavonoids might be attributed to high glucose content at T2. This finding corroborates the findings of Yang et al. [37], which showed that shikimic acid, prephenic acid and phenylpyruvic acid metabolism were strongly inhibited in dark-induced chlorotic tea plants. An increase in sugars also positively affects the glycosylation of catechins as well as the biosynthesis of flavonoid glycosides [5]. In addition, at the T1 stage, expression of genes involved in flavonoid biosynthesis was significantly downregulated, while accumulation of flavonols was increased; this may be due to a drastic increase in flavonoids causing feedback inhibition of gene expression [7].

### 4.2. The Initial Accumulation and Temperature Inhibition of Flavonols in Albino Mutant Tea Leaves Are Associated with Metabolism Related to Oxidative Stress

To survive adversity, plants modulate their metabolic systems, especially their secondary metabolism, which results in an accumulation of specific metabolites [12] such as catechins and flavones [38]. In our study, albino tea leaves accumulated flavonoids with ortho-dihydroxylated B-rings, whereas flavonoids were considerably reduced (Table 1 and Table 2). This accumulation may be attributed to their biological activity as antioxidants, because albino leaves possess unsound chloroplasts and chlorophyll deficiency, exposing them to oxidation stress. Previous studies have suggested that flavonoids such as quercetin and dihydroxy catechins serve multiple roles in the responses of higher plants to a wide range of environmental stimuli [39,40,41,42]. However, flavonoids with different molecular structures show great differences in their antioxidant activity, which is highly correlated with the substituent positions and amount of hydroxylation on the ring B group. Notably, in our study, the content of rutin, the dominant ortho-dihydroxylated compound, was not affected by greening (content did not decrease, in contrast with that of catechins, when albino leaves turned green), while it was higher in albino compared with green leaves. Furthermore, we also found that dihydroxy flavonoids were mainly distributed intracellularly in the leaf epidermal cells and the light receiving area, whereas chlorophyll and catechins were distributed throughout the cells of the chlorotic tea mutant.

## 5. Conclusions

Using metabolomics and transcriptomics to analyze a time series of leaves from a thermosensitive leaf-color mutant of tea plants, we uncovered the metabolic profile of albino tea leaves and predicted the driving factors of flavonoids in tea leaves. Flavonols accumulate under oxidative stress in albino leaves, while catechins increase with enhanced photosynthesis, indicating that biosynthesis of flavonoids might be driven by antioxidation and carbon skeleton storage under favorable and unfavorable circumstances, respectively.

## Figures and Tables

**Table 1:** Contents of main catechins, chlorophylls and carotenoids in albino and green leaves of ‘Baiye 1′ tea at the indicated time points under greening process obtained by HPLC-DAD (except total polyphenols). Different letters (a, b, c, d, e) indicate significantly different values between time points at *p* < 0.01 level (*n* = 6). NG: normal green leaf, AM: albino mutant leaf, T0: 0 day after starting greening, T1: 3 days after starting greening, T2: 6 days after starting greening.

	NG	AM	T0	T1	T2
***Catechins (µmol/mg dry weight)***					
**Gallocatechin**	10.82 ± 3.54 a	4.33 ± 0.65 c	4.57 ± 0.65 c	6.86 ± 0.33 b	11.75 ± 4.9 a
**Epigallocatechin**	22.38 ± 2.92 b	19.46 ± 3.51 b	23.35 ± 3.43 b	20.9 ± 0.16 b	26.12 ± 3.43 b
**Epigallocatechin gallate**	143.39 ± 2.42 b	98.45 ± 6.47 c	107.62 ± 8.01 c	136.26 ± 1.38 b	138.05 ± 1.18 b
**Gallocatechin gallate**	0.69 ± 0.13 b	0.43 ± 0.02 a	0.62 ± 0.03 a	0.55 ± 0.01 b	0.52 ± 0.01 b
**Catechin**	3.52 ± 0.32 a	2.01 ± 0.23 c	1.77 ± 0.06 c	1.93 ± 0.04 b	2.28 ± 0.2 a
**Epicatechin**	17.84 ± 0.71 c	14.13 ± 3.53 c	16.58 ± 4.03 c	21.2 ± 2.96 c	18.48 ± 0.45 c
**Epicatechin gallate**	23.47 ± 1.8 b	18.34 ± 1.53 d	20.29 ± 1.36 d	25.33 ± 0.23 c	27.35 ± 1.7 b
**Catechin gallate**	1.39 ± 0.15 c	0.67 ± 0.02 e	0.81 ± 0.02 e	1.13 ± 0.02 d	1.27 ± 0.05 c
**Gallocatechin**	12.82 ± 2.7 a	4.02 ± 0.39 c	4.57 ± 0.65 c	6.86 ± 0.33 b	11.75 ± 4.9 a
***Chlorophylls and carotenoids (μg/g, fresh weight)***					
***Chlorophyll* b**	240.21 ± 6.25 a	94.24 ± 7.73 c	104.36 ± 8.48 c	196.62 ± 8.61 b	255.70 ± 9.73 a
***Chlorophyll* a**	804.21 ± 26.81 b	291.25 ± 14.53 d	280.04 ± 19.92 d	664.27 ± 46.77 c	900.17 ± 40.80 a
**Lutein**	223.07 ± 15.92 a	88.87 ± 3.24 d	79.63 ± 6.13 d	143.91 ± 13.57 c	183.87 ± 17.56 b
***alpha*-Carotene**	2.39 ± 0.05 d	32.99 ± 2.45 a	30.08 ± 2.35 a	8.76 ± 0.55 b	5.61 ± 0.16 c
***beta*-Carotene**	2.98 ± 0.15 d	89.59 ± 6.92 a	80.55 ± 7.02 a	12.75 ± 0.33 b	7.98 ± 0.79 c
**Neoxanthin**	66.96 ± 3.21 b	26.84 ± 1.19 c	24.44 ± 5.39 c	71.76 ± 8.47 a	76.11 ± 9.04 a
**Violaxanthin**	87.65 ± 3.56 a	50.31 ± 1.88 c	49.34 ± 3.08 c	68.38 ± 7.66 b	77.93 ± 10.08 a
**zeaxanthin**	1.67 ± 0.04 c	3.75 ± 0.32 a	3.02 ± 0.22 a	0.97 ± 0.46 d	2.80 ± 0.55 b
***Total polyphenols and total flavonol***					
**Total polyphenols (*mg/g, dry weight*)**	215.21 ± 9.20 a	114.45 ± 11.58 c	110.55 ± 14.46 c	176.71 ± 10.06 b	234.82 ± 14.85 a
**Myricetin (*μg/g, dry weight)***	2.51 ± 0.14 a	2.55 ± 0.25 b	2.68 ± 0.20 b	2.18 ± 0.27 c	2.64 ± 0.18 a
**Quercetin (*μg/g, dry weight)***	1.25 ± 0.08 c	1.87 ± 0.05 a	1.73 ± 0.05 a	1.49 ± 0.04 b	1.18 ± 0.06 c
**Kaempferol (*μg/g, dry weight)***	1.52 ± 0.10 b	1.66 ± 0.04 a	1.75 ± 0.11 a	1.42 ± 0.07 b	1.44 ± 0.07 b

**Table 2:** Putatively identified secondary metabolites, analyzed by LC-MS, showing significant changes in their relative abundance in young shoots of tea plants at the indicated time points under greening process.

	AM/NG			T1/T0			T2/T0			T2/T1		
Name	LOG2fold	VIP	*p*	LOG2fold		*p*	LOG2fold	VIP	*p*	LOG2fold	VIP	*p*
Epiafzelechin 3-gallate	−1.11	1.40	*	1.33	1.76	*	1.42	1.69	*	0.08	0.54	
Epigallocatechin 3-cinnamate	−2.08	1.41	*	−0.37	0.89		0.88	1.52	*	1.26	1.75	*
Epigallocatechin 3-coumaroate	−1.76	1.49	*	0.18	1.33	*	1.26	1.68	*	1.08	1.80	*
3 Hydroxycoumarin	−1.45	1.05	*	0.18	0.34		1.17	1.31	*	0.99	1.38	*
epicatechin 7-O-glucuronide	−3.11	1.45	*	0.43	1.08	*	0.96	1.49	*	0.53	1.40	*
Apigenin glucoside arabinoside	−1.90	1.37	*	0.00	0.03		1.39	1.67	*	1.38	1.79	*
Cyanidin 3-rutinoside	−3.54	1.19	*	2.22	1.68	*	2.57	1.60	*	0.35	0.94	
Delphinidin coumaroylglucoside)	−3.38	1.47	*	2.10	1.74	*	2.51	1.66	*	0.40	1.28	*
Gladiatoside C2	−6.79	1.48	*	2.39	1.54	*	2.90	1.59	*	0.50	1.01	
Glycitin	−1.71	1.43	*	1.60	1.67	*	2.01	1.67	*	0.41	1.26	*
Hyacinthin	−5.23	1.16	*	2.64	1.55	*	2.91	1.53	*	0.27	0.56	
Isovitexin glucoside	−1.15	1.50	*	1.68	1.83	*	1.97	1.68	*	0.29	1.49	*
Naringin	−2.39	1.50	*	0.17	0.98		0.53	1.58	*	0.36	1.70	*
Vanillic acid	−1.41	1.43	*	0.29	0.64		0.74	1.42	*	0.46	1.03	
Cyanidin 3-glucoside	1.67	1.43	*	−0.02	0.07		−1.17	1.52	*	−1.15	1.63	*
Kaempferol glucopyranoside	1.67	1.46	*	−0.60	1.57	*	−1.22	1.61	*	−0.62	1.69	*
Rutin(Quercetin 3-Rutinoside)	2.98	1.50	*	−1.04	1.64	*	−1.86	1.62	*	−0.82	1.32	*
Myricetin 3-arabinoside	1.14	1.51	*	−0.90	1.71	*	−1.77	1.65	*	−0.87	1.73	*
Naringenin glucuronide	2.22	1.43	*	−0.67	1.57	*	−1.84	1.63	*	−1.17	1.83	*
Quercetin 4′,7-diglucoside	1.76	1.45	*	−2.08	1.78	*	−2.80	1.65	*	−0.72	1.02	*

“*” indicates significant difference at *p* < 0.001 level; NG: normal green leaf, AM: albino mutant leaf, T0: 0 day after starting greening, T1: 3 days after starting greening, T2: 6 days after starting greening.

**Table 3:** Putatively identified primary metabolites, analyzed by GC-MS, showing significant changes in their relative abundance in young shoots of tea plants at the indicated time points under greening process.

	AM/NG	T1/T0	T2/T0	T2/T1
***Carbohydrate***				
D-Arabinopyranose	−1.21 *	0.53 *	1.22 *	0.69 **
d-Galactose	−1.28 **	0.10	0.29	0.19
D-Glucitol	−3.84 **	0.39	0.76 *	0.37
d-Glucose	−0.43 **	0.61 **	0.33	0.28 *
D-Psicose	−0.67 **	−0.17 *	−0.24 *	−0.07
Mannose	0.26	0.33	0.71 **	0.38 *
Inosose	−2.65 **	1.06 **	0.56	−0.50 *
L-(-)-Sorbose	−0.59 **	0.18	−0.17	0.00
***Amino acid***				
l-Glutamine	0.80 **	−2.09 **	−3.34 **	−1.25 *
L-Proline	1.53 **	0.08	−2.24 **	−2.32 *
L-Theanine	1.53 **	−0.23 *	−0.80 *	−0.57 *
L-Aspartic acid	1.60 **	−0.33 *	−1.22 **	−0.89 *
L-Glutamic acid	1.63 **	−0.56 **	−1.69 **	−1.13 *
Serine	1.94 **	−0.56 *	−1.44 **	−0.88 *
Asparagine	0.56	1.46 **	−0.18	−1.64 *

Value is log2 (fold change); “*” and “**” indicate significant difference at *p* < 0.01 or *p* < 0.001 level; NG: normal green leaf, AM: albino mutant leaf, T0: 0 day after starting greening, T1: 3 days after starting greening, T2: 6 days after starting greening.

**Table 4:** Genes identified by weighted gene co-expression network analysis (WGCNA).

Gene ID	Annotation	Class_Annotation
CSS0035261	ADP glucose pyrophosphorylase	Starch and sucrose metabolism
CSS0026029	photosystem II protein H (chloroplast)	Photosystem II reaction center protein
CSS0032826	starch synthase 1, chloroplastic/amyloplastic-like	Starch and sucrose metabolism
CSS0004941	UDP-Glycosyltransferase superfamily protein isoform 1	Starch and sucrose metabolism
CSS0025971	ferredoxin-3, chloroplastic	Photosynthesis
CSS0009596	hypothetical protein PRUPE_ppa020609mg, partial	Defense mechanisms
CSS0040654	NF-X1-type zinc finger protein NFXL1-like	response to stress | response to abiotic stimulus
CSS0019730	peroxidase 12	peroxidase activity | response to oxidative stress

## Data Availability

All data are available in this publication.

## Supplementary Materials

The following supporting information can be downloaded at: https://www.mdpi.com/article/10.3390/molecules27185792/s1, Figure S1. The chromatogram obtained by LC-DAD; Figure S2. Pathway related to the different metabolites between albino and green tea leaves of baiye1; Table S1. Primer sequences for quantitative RT-PCR; Table S2. Functional classification of differentially expressed genes using Gene Ontology; Table S3. Enrichment of differentially expressed genes using KEGG. Table S4. Retention time and mass spectra data that were the basis for the identification of the compounds by LC-MS.
