# Supplementary material for: Integration of Metabolomics and Transcriptomics Reveal the Mechanism Underlying Accumulation of Flavonols in Albino Tea Leaves
Source: Molecules. 2022 Sep 7;27(18):5792. doi: 10.3390/molecules27185792 (PMC9501457; doi:10.3390/molecules27185792)
Supplement: Supplementary file 1 [file molecules-27-05792-s001.zip › molecules-1791681-supplementary.pdf]

molecules

MDPI

Supplementary Materials

Integration of Metabolomics and Transcriptomics Reveal the Mechanism Underlying Accumulation of Flavonols in Albino Tea Leaves

Qunfeng Zhang [1], Chunlei Li [2], Zixin Jiao [1], Jianyun Ruan [1] and Mei-Ya Liu [1,*]

[1] Key Laboratory for Plant Biology and Resource Application of Tea, Tea Research Institute, Chinese Academy of Agricultural Sciences, The Ministry of Agriculture, Hangzhou 310058, China
[2] Weifang University of Science and Technology, Shandong Provincial University Laboratory for Protected Horticulture, Shouguang 262700, China
* Correspondence: liumeiya@tricaas.com; Tel.: +86-571-86653859

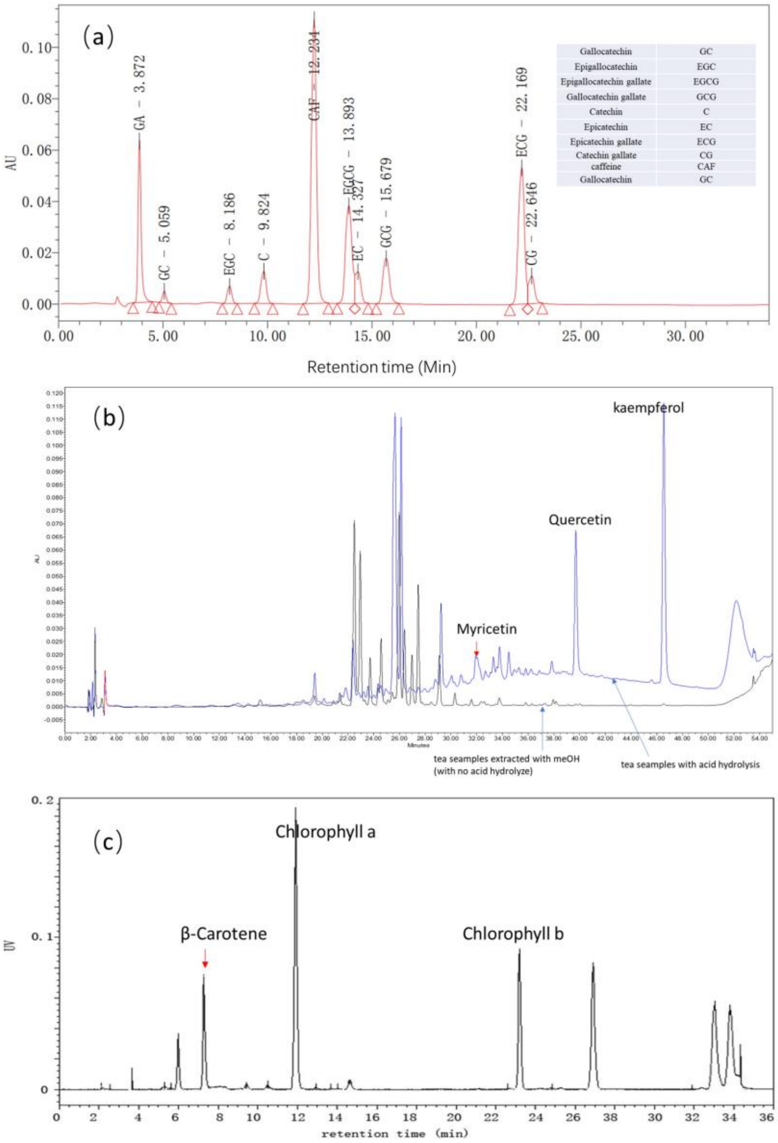

Figure S1. Figure S1 The chromatogram of catechins (**a**), flavonols (**b**), and chlorophyll (**c**) obtained by LC-DAD.

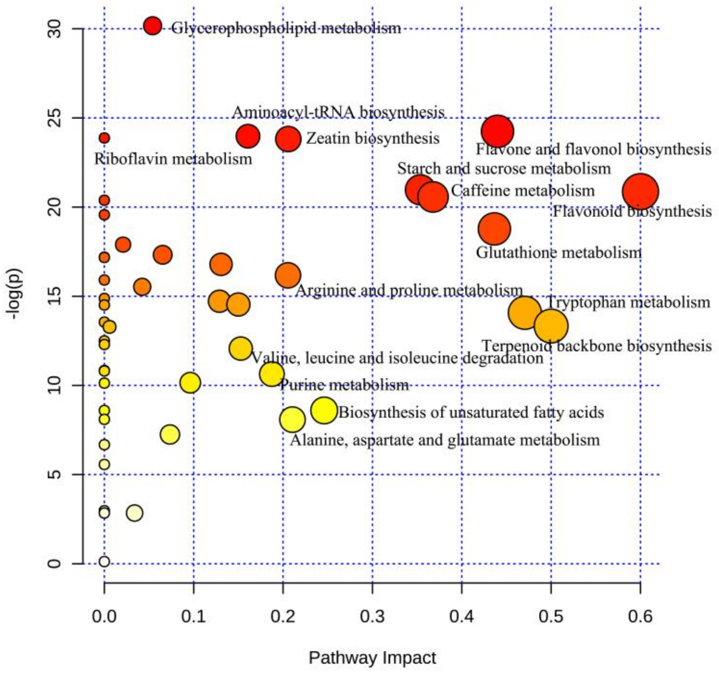

Figure S2. Pathway relatied to the different metabolites between albino and green tea leaves of baiye1.

Table S1. Primer sequences for quantitative RT-PCR.

Genes	Forward Primer (5′ - 3′)	Reverse Primer (5′- 3′)
4CL	CGTGGTCCTCAAATTATG	CATCATCGTCGTCTACAT
ANR	GCGAAGTTGATCCTCTCGTC	AACCACATCGTCAAGTGAACA
ANS	TAATGGCAAGTACAAGAG	CAATGGCTTCAAGATAATC
C4H	CAATGGCAATGACTTTAG	CTCAGCAGTATCAATCTT
CHI	GTGATGGATGAAGTTGTG	AAGAGAGAAAGCAGAGTC
CHS	TTACTAATAGCGAGCATAAGGT	CTAGCATCAAGCGAAGGT
DFR	AGTTGTGTCGTTCTCATC	GTATCAATGGCTCCTCTG
F3′5′H	AATCCTGGTGAAGAGAAG	TCTATTATGCTTGATGATGTG
F3′H	ACCTTTCGACTTCACCCATCAAC	TAACTGGACCATACGCAACCCTA
F3H	GCGACAGTATACCCCCTGAA	AGTATGGCAAAGGCACATCC
FLS	GGAGAACAGCAAGGATATCG	TCTCCTCCTGTGGGAGCTTA
LAR	GGGGCATCCTGTATCAAAGA	CCGCATACCTTTCAGTCCAT
PAL	ATGACTTCTACAACAATGG	GGAGTTCTGAGCAATAAG
GAPDH	TTGGCATCGTTGAGGGTCT	CAGTGGGAACACGGAAAGC

4Cl, 4-coumarate--CoA ligase; ANR, anthocyanidin reductase; ANS, anthocyanidin synthase; C4H, cinnamate 4-hydroxylase; F3′H, flavonoid-3'-hydroxylase; PAL, Phenylalanine ammonia-lyase; CHI, chalcone isomerase; CHS, Chalcone synthase; F3H, flavonoid-3-hydroxylase; LAR, leuco-anthocyanidin reductase; FLS, flavonol synthase; DFR, dihydroflavonol-4-reductase; F3′5′H, flavonoid-3', 5'-hydroxylase.

Table S2. Functional classification of differentially expressed genes using Gene Ontology.

iD	Term	Genes [a]	p	FDR	ES[b]
	AL/NG				
GO:0005618	cell wall	28	0.001	0.009	1.842
GO:0005199	structural constituent of cell wall	5	0.000	0.002	6.859
GO:0004601	peroxidase activity	13	0.000	0.004	2.903
GO:0030093	chloroplast photosystem I	2	0.000	0.004	15.862
GO:0009535	chloroplast thylakoid membrane	39	0.000	0.000	2.447
GO:0042742	defense response to bacterium	39	0.000	0.001	2.038
GO:0009523	photosystem II	6	0.000	0.002	6.261
GO:0055114	oxidation-reduction process	207	0.000	0.000	1.502
GO:0006979	response to oxidative stress	28	0.000	0.005	1.997
GO:0000322	storage vacuole	2	0.000	0.000	47.973
	T0/T1				
GO:0004089	carbonate dehydratase activity	6	0.000	0.001	7.435
GO:0009011	starch synthase activity	5	0.000	0.002	6.837
GO:0016036	cellular response to phosphate starvation	18	0.000	0.000	3.800
GO:0009544	chloroplast ATP synthase complex	1	0.000	0.000	51.092
GO:0055114	oxidation-reduction process	118	0.000	0.007	1.367
GO:0010282	senescence-associated vacuole	3	0.000	0.002	13.934
GO:0006950	response to stress	23	0.000	0.006	2.186
GO:0009569	chloroplast starch grain	1	0.000	0.007	23.166
GO:0047213	anthocyanidin 3-O-glucosyltransferase activity	14	0.000	0.001	2.972
GO:0030247	polysaccharide binding	25	0.000	0.000	7.097
	T2/T1				
GO:0080002	UDP-glucose:4-aminobenzoate acylglucosyltransferase activity	5	0.000	0.001	8.884
GO:0080043	quercetin 3-O-glucosyltransferase activity	10	0.000	0.000	7.361
GO:0080044	quercetin 7-O-glucosyltransferase activity	8	0.000	0.000	7.928
GO:0043481	anthocyanin accumulation in tissues in response to UV light	35	0.000	0.000	5.185
GO:0005975	carbohydrate metabolic process	60	0.000	0.000	2.280
GO:0052689	carboxylic ester hydrolase activity	19	0.000	0.000	3.040
GO:0010413	glucuronoxylan metabolic process	35	0.000	0.000	3.930
GO:0006950	response to stress	20	0.001	0.010	2.068
GO:0009523	photosystem II	8	0.000	0.000	6.525
GO:0005776	autophagic vacuole	10	0.001	0.013	10.306

a, numbers of significantly changed gene, b, ES: enrichment score.

Table S3. Enrichment of differentially expressed genes using KEGG.

iD	Term	ListHits	p	FDR	Enrichment_score
**ko04110**	**Cell cycle**	16	0.00	0.00	3.12
**ko03010**	**Ribosome**	20	0.00	0.03	1.94
**ko00500**	**Starch and sucrose metabolism**	15	0.00	0.01	2.75
**ko00195**	**Photosynthesis**	6	0.01	0.03	2.78
**ko00942**	**Anthocyanin biosynthesis**	1	0.00	0.02	13.22
**ko00942**	**Anthocyanin biosynthesis**	1	0.00	0.01	12.83
**ko00710**	**Carbon fixation in photosynthetic organisms**	7	0.00	0.10	2.63
**ko00940**	**Phenylpropanoid biosynthesis**	6	0.00	0.02	2.93
**ko00944**	**Flavone and flavonol biosynthesis**	2	0.00	0.03	5.63
**ko00360**	**Phenylalanine metabolism**	11	0.00	0.00	3.26
**ko00190**	**Oxidative phosphorylation**	15	0.00	0.00	4.16
**ko00940**	**Phenylpropanoid biosynthesis**	7	0.01	0.06	2.63
**ko00195**	**Photosynthesis**	8	0.00	0.03	3.95
**ko00940**	**Phenylpropanoid biosynthesis**	8	0.00	0.03	2.82
**ko00941**	**Flavonoid biosynthesis**	7	0.00	0.00	4.29

Table S4. Retention time and mass spectra data that were the basis for the identification of the compounds by LC-MS.

name	Mass Error (ppm)	Isotope Similarity	m/z	Retention time (min)
Epiafzelechin 3-gallate	−0.67	87.67	426.0951	4.062217
Epigallocatechin 3-cinnamate	−1.74	95.55	436.1158	8.125367
Epigallocatechin 3-p-coumaroate	−4.12	99.20	452.1107	6.376683
3 Hydroxycoumarin	−6.70	98.36	162.0317	6.240967
epicatechin 7-O-glucuronide	6.29	94.00	466.1111	5.686633
Apigenin 6-C-glucoside 8-C-arabinoside	2.70	92.12	564.1479	5.643783
Cyanidin 3-rutinoside	−6.63	98.59	630.1351	4.502267
Delphinidin 3-(3''-p-coumaroylglucoside)	1.28	90.07	611.1401	4.2451
Gladiatoside C2	4.46	92.17	566.1424	5.658067
Glycitin	−4.27	95.19	446.1213	6.369533
Hyacinthin	5.96	99.44	120.0575	4.480833
Isovitexin 2''-O-glucoside	0.72	97.20	594.1585	4.266533
Naringin	−0.72	92.44	580.1792	6.155233
Vanillic acid	2.98	93.11	168.0423	3.207883
Cyanidin 3-glucoside	2.20	93.86	484.0772	2.860717
Kaempferol 3-β-d-glucopyranoside	−4.30	96.14	448.1005	5.658067
Rutin	1.80	91.22	610.1534	4.6137
Myricetin 3-arabinoside	−4.53	96.53	450.0798	2.796433
naringenin-4'-O-glucuronide	−0.21	98.07	448.1006	6.1338
Quercetin 4',7-diglucoside	−8.22	94.22	626.1483	3.9908
